# Impact of COVID-19 Pandemic Perception on Job Stress of Construction Workers

**DOI:** 10.3390/ijerph191610169

**Published:** 2022-08-17

**Authors:** Huakang Liang, Tianhong Liu, Wenqian Yang, Fan Xia

**Affiliations:** School of Economics and Management, Beijing Jiaotong University, Beijing 100044, China

**Keywords:** COVID-19, pandemic perception, job stress, problem-focused coping, emotion-focused coping, construction workers

## Abstract

Construction has been regarded as one of the most stressful industries, and the COVID-19 pandemic has deteriorated this situation. This research developed and tested a model of the impact of COVID-19 pandemic perception on job stress of construction workers. Both problem-focused and emotion-focused coping were considered as mediators. Empirical data were collected using a detailed questionnaire from the Chinese construction industry. The results showed that pandemic perception was significantly related to psychological and physical stress. Emotion-focused coping was mainly triggered by pandemic fear and job insecurity, while problem-focused coping was mainly triggered by organizational pandemic response. Furthermore, the effects of pandemic fear and organizational pandemic response on job stress were mediated by problem-focused coping. Finally, the theoretical and practical significance, research limitations, and future research directions of this study are discussed.

## 1. Introduction

The COVID-19 pandemic has impacted most industries globally, including the construction industry [1]. The construction industry is vulnerable to the COVID-19 crisis because construction workers need to operate closely within a limited space with many nodes of activities concurrently [2]. Previous investigations have revealed that compared with general industries (e.g., healthcare, manufacturing, and transportation), construction workers reported the highest number of positive cases [3,4]. Apart from the high infection risk, this pandemic has brought unprecedented economic impacts on the construction industry due to labor shortages, suspension, and cancellation of projects, and disrupted supply and logistics [5]. For instance, the average unemployment rate of construction workers in the USA increased by 95%, from 4.5% in 2019 to 8.7% in 2020 [6]. Therefore, COVID-19 has introduced significant challenges for frontline construction workers, such as increased concerns about economic insecurities and well-being, as well as the implementation of new and changing guidance in order to reduce the spread of the virus [7].

The impacts of the COVID-19 pandemic on the construction industry have attracted much attention recently. For instance, Ling et al. (2022) revealed that construction projects suffered significant delays, cost overruns, and lower quality during the pandemic [8]. Al-Mhdawi et al. (2022) found that the pandemic impacts involved contractual implications, the construction financial market, supply chain operations, and safety and risk management [9]. Jeon et al. (2022) proposed the Purdue Index to assess the impact of COVID-19 pandemic on the American construction industry [10]. Furthermore, some COVID-19-related studies have tended to explore strategies for pandemic impacts. For instance, Raoufi and Fayek (2022) suggested best practices for construction organizations to control and mitigate pandemic challenges [11]. Goh et al. (2022) proposed a smart real-time monitoring system to ensure safe distancing during workers’ daily activities [12]. However, previous studies have primarily focused on the economic, financial, and operational consequences of the COVID-19 pandemic, while few have investigated the occupational health and safety of construction workers during the pandemic [13]. The adverse influence of the COVID-19 pandemic on occupational stress has been well-recognized in other high-risk industries, such as healthcare [14,15] and food service [16]. Despite the similarity, construction industry has its unique characteristics such as high mobility of a temporary workforce, fragmented and pervasive subcontracting system, and un-repeated and complex project environments [17]. Therefore, it is necessary to further examine the influence of COVID-19 on the occupational stress of construction workers.

Construction is generally accepted as one of the most stressful industries due to physically and mentally demanding tasks performed in a high-risk environment [18]. It was reported that 68% of construction workers are under excessive pressure to work in the construction industry [18]. However, COVID-19 will further deteriorate such stressful situations. On the one hand, worse finance and unemployment raised by delayed and suspended construction projects would exacerbate the job insecurity of construction workers [2]. On the other hand, the high infection rate and relatively high mortality rate of COVID-19 also cause fear and panic among construction workers. These, combined with the unique characteristics of the construction industry, would lead to poor psychological and physical stress of construction workers [2].

Furthermore, while previous studies have demonstrated the adverse effects of COVID-19 pandemic on occupational stress, little is known about the mediating mechanism underlying this relationship. Coping theory argues that merely facing various sources of stress at work does not matter, but inappropriate coping behaviors can lead to the generation of stress symptoms [19,20]. Coping behaviors represent individual responses to stressful situations [20]. Therefore, this research would examine the mediating roles of coping behaviors between pandemic perception and occupational stress among construction workers. The research findings would shed some lights on creating health intervention programs to promote the health and well-being of construction workers during and after the COVID-19 pandemic.

## 2. Literature Review and Hypotheses Development

### 2.1. COVID-19 Pandemic Perception and Job Stress

Pandemic perception represents the ways in which construction workers perceive the stressful situations induced by the current pandemic, mainly including pandemic fear, organizational pandemic response, and job insecurity [13,21,22]. Pandemic fear refers to the individual perceived risks of being infected by COVID-19 as well as other health consequences (e.g., the high mortality rate). Job insecurity reflects individual concerns about the prospect of job continuity due to the sluggish economy that has been triggered by the pandemic. Organizational pandemic response captures whether organizational responses are adequate to protect workers from the current pandemic.

Fear is known as one of the most primitive feelings when facing the real or perceived threats [23]. The COVID-19 pandemic has caused exceptionally high levels of fear due to its worldwide spread, high media attention, lack of public knowledge and effective medical treatment, and drastic and unprecedented preventive measures (e.g., lockdowns and quarantine) [24]. The construction industry is no exception. The construction industry is highly susceptible to COVID-19 due to the physical presence of a large on-site workforce within limited space as well as the mobility of workers constantly moving across projects [25]. The fears related to the COVID-19 pandemic among construction workers can be presented in various ways, such as fear of contracting the virus, fear about family members’ health, and fear of the possible adverse socioeconomic consequences of the pandemic [26,27].

Although fear can be considered as an adaptive emotion to cope with potential threats, it may eventually contribute to growing levels of stress [28,29]. Stress is workers’ response to the imbalance between the demands of external events and the resources to handle the demands [30]. Construction workers usually suffer from a lot of types of stress, including physical and psychological, where the former may lead to problems such as sleep disorders, headaches, and skin problems, while the latter can induce anxiety, sadness, anger, and tension in the workplace [31]. According to the conservation of resources theory [32], during the pandemic, construction workers may have to invest psychological and physical resources to cope with their fear of COVID-19, which will lead to resource loss to handle both physical and psychological stress.

**Hypothesis** **1a.** *Pandemic fear will be positively related to individuals’ psychological stress.*

**Hypothesis** **1b.** *Pandemic fear will be positively related to individuals’ physical stress.*

Perception of job insecurity, namely, worry about job continuation, has been regarded as another key stressor due to the economic shocks caused by COVID-19 [33]. In the USA, about 40% of construction companies had been laid off due to the cancellations of contracts and scarcity of machinery or supplies triggered by the outbreak [7]. The construction industry in the UK also had experienced a 7.7% reduction in the workforce [1]. The high rate of unemployment in the construction industry could increase workers’ concerns about job insecurity, which might damage well-being and health, and create anxiety, stress, and depression. Apart from this, job insecurity may incite employees to invest additional efforts in an attempt to maintain existing positions. Such further efforts by employees may lead to adverse psychological, behavioral, and emotional feelings that exacerbate their emotional exhaustion [34].

**Hypothesis** **2a.** *Job insecurity will be negatively related to individuals’ psychological stress.*

**Hypothesis** **2b.** *Job insecurity will be negatively related to individuals’ physical stress.*

The construction industry is highly susceptible to COVID-19 due to the physical presence of a large on-site workforce within limited space [25]. As the world emerges from lockdown, the construction industry must learn how to balance productivity and safety to protect its workers from the threat of a new wave [13]. Organizational positive responses to COVID-19, such as the strict enforcement of social distancing, field disinfection, and health training, may inform workers that the organization has a positive orientation towards their health and well-being. Based on the social exchange theory [35], individuals respond accordingly to how they perceive they are treated by their organization. Being satisfied with organizational pandemic response would motivate workers to benefit organizations in return that contribute to the reduction in job stress [36]. Conversely, when they view the organization’s COVID-19 responses in a negative manner, workers are less likely to trust the organization regarding pandemic prevention that causes a higher stress level. Therefore, the hypotheses are as follows:

**Hypothesis** **3a.** *Organizational pandemic response will be positively related to individuals’ psychological stress.*

**Hypothesis** **3b.** *Organizational pandemic response will be positively related to individuals’ physical stress.*

### 2.2. Mediating Roles of Coping Strategies

Coping refers to an individual’s constantly changing cognitive and behavioral efforts in managing one’s external and/or internal stimuli that are higher than one’s own resources [37,38]. In general, two types of coping behaviors are identified: problem-focused and emotion-focused [19]. Problem-focused coping is proactive and aims at addressing the problem to decrease or eliminate stress (e.g., information-seeking planning, or taking actions). By contrast, emotion-focused coping is reactive and refers to attempting to regulate feelings and emotional responses to the stressful situation rather than deal with the problem itself (e.g., seeking emotional support from others) [39].

Pandemic fear could affect individuals’ behavior and how to intuitively assess the threat they are exposed to [23]. Workers in panic tend to overestimate difficulties and cope with stressful situations in a passive manner. They will be reluctant to address the source of stress directly but put more effort into regulating their negative emotions. Moreover, a previous study showed that in the early stage of pandemic, people’s sense of control was threatened by incomplete information of virus, involuntary changes in behaviors, apparent financial difficulties, etc. [40]. The low sense of control induced by pandemic fear may also be related to emotion-focused coping behaviors [41]. From this standpoint, we argue that pandemic fear will increase emotion-focused coping but hinder problem-focused coping.

**Hypothesis** **4a.** *Pandemic fear will be negatively related to individuals’ problem-focused coping behaviors;*

**Hypothesis** **4b.** *Pandemic fear will be positively related to individuals’ emotion-focused coping behaviors.*

Previous studies have revealed that job insecurity may stimulate negative attitudes and behaviors among workers [36,42]. When individuals are uncertain about the future of their jobs, they tend to withdraw emotionally and behaviorally [36]. Job insecurity may deplete individuals’ valued resources such as stable income and development opportunities, and they will have low motivation to spend more time and energy dealing with the threat proactively. In addition, individuals with a high sense of job insecurity tend to feel powerless and uncertain about their working environment, and thus they are more likely to adopt negative coping strategies to reduce risks and protect themselves [43,44]. Based on this reasoning, we propose the following hypotheses.

**Hypothesis** **5a.** *Job insecurity will be negatively related to individuals’ problem-focused coping behaviors;*

**Hypothesis** **5b.** *Job insecurity will be positively related to individuals’ emotion-focused coping behaviors.*

Individual coping strategies mainly depend on the available resources and constraints. Organizational pandemic response could not only provide necessary resources (e.g., masks and other personal protective equipment), but also contribute to developing individual abilities and strengths to cope with adverse pandemic impacts. In other words, when workers feel satisfied with the organizational responses towards the pandemic, they tend to believe that they have available resources and are more likely to use problem-focused coping such as seeking the help of managers or relying on personal strength to explore new stress coping strategies [36,45,46]. As a result, with an adequate organizational response during and after the pandemic, individuals might adopt more problem-focused coping and less emotion-focused coping.

**Hypothesis** **6a.** *Organizational pandemic response will be positively related to individuals’ problem-focused coping behaviors;*

**Hypothesis** **6b.** *Organizational pandemic response will be negatively related to individuals’ emotion-focused coping behaviors.*

Coping styles are related to the process in which stress occurs, and different coping styles will bring different degrees of job stress to individuals [39]. Specifically, problem-focused coping could alleviate both physical and psychological stress through eliminating the sources of stress, while emotion-focused coping would deteriorate the stress symptoms [37,47]. As mentioned earlier, pandemic perception may have significant effects on coping behaviors, which in turn predict workers’ physical and psychological stress. Therefore, we propose the following hypotheses.

**Hypothesis** **7a.** *Problem-focused coping mediates the relationship between pandemic fear and job stress;*

**Hypothesis** **7b.** *Emotion-focused coping mediates the relationship between pandemic fear and job stress;*

**Hypothesis** **8a.** *Problem-focused coping mediates the relationship between job insecurity and job stress;*

**Hypothesis** **8b.** *Emotion-focused coping mediates the relationship between job insecurity and job stress;*

**Hypothesis** **9a.** *Problem-focused coping mediates the relationship between organizational pandemic response and job stress;*

**Hypothesis** **9b.** *Emotion-focused coping mediates the relationship between organizational pandemic response and job stress.*

**Figure 1:**
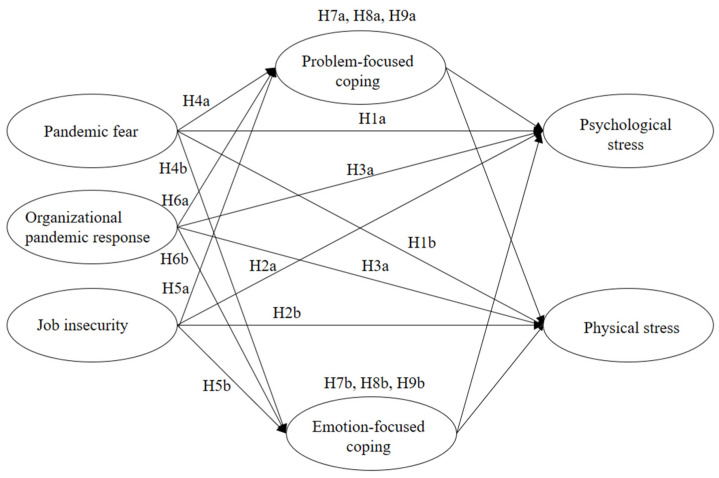
The hypothetical model (H1a–H9b) proposed by this research.

The hypothesized theoretical model is summarized in Figure 1. This model posits that, through problem-based coping and emotion-based coping, pandemic perception (represented by pandemic fear, job insecurity, and organizational pandemic response) has an impact on occupational stress (represented by physical stress and psychological stress) of construction workers.

## 3. Method

A pilot study was conducted before the formal survey in order to check whether the questionnaire was applicable. A total of 10 workers participated in this pilot, based on which the initial questionnaire was revised.

### 3.1. Measures

#### 3.1.1. Pandemic Fear

The questions for assessing pandemic fear were adapted from the items validated by Chi et al. (2020) [48]. Sample items include “Currently, the treatment methods for COVID-19 are not effective” and “I am worried about myself, my family members or my colleagues who may be affected by COVID-19”.

#### 3.1.2. Organizational Pandemic Response

The questions about organizational pandemic response were based on the study of Watkins et al. (2015) [49]. It was measured with three items that described construction workers’ satisfaction with the pandemic measures taken by organizations. Sample items include “I am satisfied with the way that my project responded to COVID-19” and “My project did everything that it could have in response to COVID-19”.

#### 3.1.3. Job Insecurity

The questions about job insecurity were adapted from the study of Tang et al. (2020) [50]. It was measured with three items that described how insecure construction workers felt about their current jobs. Sample items include “The COVID-19 pandemic makes me feel that my job prospects will change” and “COVID-19 makes me feel that my work is unstable”.

#### 3.1.4. Coping Behaviors

The questions related to coping behaviors referred to the study of Liang et al. (2021) [51] and Srivastava and Tang (2018) [52]. Problem-focused coping was measured with three questions that describe efforts to directly change problematic situations. Sample items include “When encountering a stressful situation, I will try to get advice from someone about what to do” and “When encountering a stressful situation, I will try to get support from colleagues, friends or relatives”. By contrast, emotion-focused coping was measured with three questions about how individuals regulate the emotional distress associated with the situation. Sample items are “When encountering a stressful situation, I will use alcohol or tobacco to make myself feel better” and “When encountering a stressful situation, I will pretend that the problem hasn’t really happened”.

#### 3.1.5. Job Stress

The questions about job stress were based on the studies of Leung et al. (2012) [53] and Leung et al. (2017) [54]. Psychological stress was measured with three questions to capture negative emotional states of construction workers. Sample items are “I often feel nervous in the workplace due to issues at work” and “I often feel sad in the workplace due to issues at work”. By contrast, physical stress was measured with four questions regarding the physiological reaction of the human body. Sample items include “I often have headaches and migraines” and “I often have skin problems such as skin irritations and skin disorders”.

### 3.2. Participant and Questionnaire Administration

The final questionnaire included five demographic questions that were about respondents’ gender, age, work experience, education level, and marital status. Twenty-four health-specific questions were developed with the aim of collecting meaningful data about the hypothesized relationships in Figure 1. The questions were scored using a five-point Likert rating scale, with answers ranging from one (strongly disagree) to five (strongly agree).

Between January and April 2021, the formal paper questionnaires were distributed to construction workers in twenty-one construction projects in six different cities in China. These projects were selected based on previous working relationships as well as the permission of the corporate-level managers. The questionnaires, with a cover letter explaining the purposes and procedure of the study, were delivered to those workers. Those who agreed to participate were assured of anonymity and that their participation would be voluntary. With the help of senior management, the questionnaire was administrated to a total of 800 construction workers in 2021. The final sample consisted of 498 valid responses because of unrecovered and incomplete questionnaires (a response rate of 62.25%).

**Figure 2:**
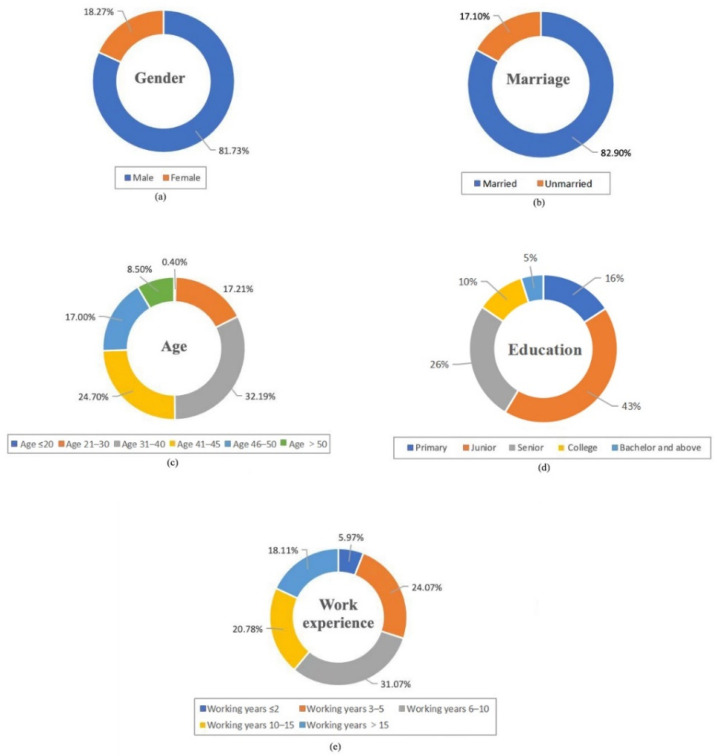
Descriptive statistics in terms of (**a**) gender; (**b**) marriage; (**c**) age; (**d**) education; (**e**) work experience.

Figure 2 shows the demographic characteristics of the respondents. The majority of the respondents were male (81.73%), because of the male-dominant workforce in the Chinese construction industry. Respondents were primarily married, accounting for 82.90%. Among the respondents, 31–40 years old and 41–45 years old accounted for the largest proportions (32.19% and 24.70%, respectively). A majority of respondents (85%) had only completed primary, junior, or senior education, indicating a low level of education among Chinese construction workers. Most workers had been working in the construction industry for more than five years (70.5%).

### 3.3. Statistical Procedures

Based on the Maximum Likelihood Estimation (MLE), a two-stage structural equation model (SEM), including a measurement model and a structural model, was used to test the validity of the measurement model and the hypothesis of the impact of pandemic perception on job stress. Firstly, the reliability and validity of the questionnaires were analyzed using the measurement model assessment. Secondly, the path analysis method was used to estimate the structural model and test the hypotheses. The assessment of the model fit was revealed by the ratio of model chi-square to the degree of freedom ($\chi^{2}/df$), the root mean square error of approximation (RMSEA), the Incremental Fit index (IFI), the Tucker–Lewis index (TLI), and comparative fitting index (CFI) [55]. The $\chi^{2}/df$ value was 3.0 or lower, RMSEA value was 0.08 or lower, and CFI, TLI, and IFI values were 0.90 or higher, indicating a good fit [55,56,57]. All analyses were conducted using Analysis of Moment Structures (AMOS) v21.0 and SPSS v18.0 (IBM, Armonk, NY, USA).

## 4. Analysis and Results

### 4.1. Measurement Model Assessment

The reliability and validity of constructs were tested using measurement model assessment. Cronbach’s Alpha coefficient was used as a lower bound of the reliability with the value of 0.7 as cutoff, which reflects correlations between measurement items in one dimension [58,59]. Secondly, two measures of construct validity, convergent and discriminant validity, were tested, respectively [60]. Discriminant validity assesses the degree to which one construct differs from others, while convergent validity evaluates the extent to which a latent construct’s question items are highly correlated. It is recommended that the standardized factor loading (FL) value is 0.50 or higher, a composite reliability (CR) value is 0.70 or higher, and an average variance extracted (AVE) value is 0.50 or higher to achieve convergent validity. Moreover, the square roots of AVEs are recommended to be higher than the correlations between the constructs [61,62,63].

First, the result of confirmatory factor analysis was $\chi^{2}/df$ = 2.644; IFI = 0.937; TLI = 0.937; CFI = 0.947; RMSEA = 0.058, which meant an acceptable goodness-of-fit. The FL values of all measurement items were statistically significant at the level of 0.001. In terms of validity verification, as shown in Table 1, the Cronbach’s Alpha values were all greater than 0.70, suggesting a strong internal consistency reliability. All CR values were greater than 0.70 and all FL values were higher than 0.5. Almost all AVE values were greater than the 0.50 threshold, except for pandemic fear, while the FL values of its items were all higher than 0.50, and its CR value was higher than 0.70, and thus pandemic fear could also be acceptable [64]. These results proved the convergence of constructs. In addition, the square root of AVE of any two constructs was greater than the correlation between their constructs, confirming the discriminant validity of all constructs. As shown in Table 2, all the correlation values did not exceed the threshold 0.90, indicating no multi-collinearity [63]. In addition, the variance inflation factor (VIF) values were further examined using linear regression, and all VIF values (1.12–1.49) did not exceed 5 [63]. Therefore, there was no multi-collinearity between the variables.

### 4.2. Structural Model Assessment

**Figure 3:**
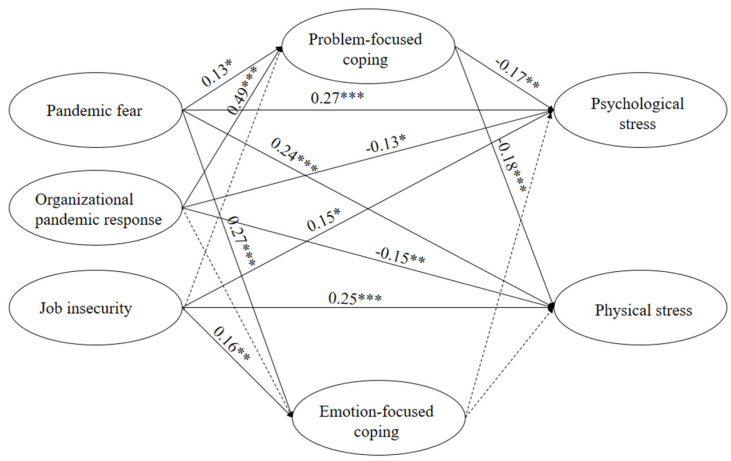
Estimated structural model ($\chi^{2}/df=2.641;\mathrm{IFI}=0.947;\;\mathrm{TLI}=0.937;\;\mathrm{CFI}=0.947;\;\mathrm{RMSEA}=0.057$). Note: *** *p* < 0.001, ** *p* < 0.01, * *p* < 0.05, n.s. *p* > 0.05. Solid line: path coefficient is statistically significant; dashed line: path coefficient is not statistically significant.

Figure 3 shows the estimated structural model and standardized path coefficients. The fitting index measures ($\chi^{2}/df=2.641;$ IFI = 0.947; TLI = 0.937; CFI = 0.947; RMSEA = 0.057) were all within the recommended range, indicating that the hypothetical model fits well with the empirical data.

As shown in Figure 3, a significant standardized path coefficient in the hypothesized direction suggests that the corresponding hypothesis can be verified. Specifically, Hypothesis 1 proposed that pandemic fear would be positively related to job stress. Hypothesis 1 was supported because the two paths between pandemic fear and physical stress (β=0.24;p<0.001) and psychological stress (β=0.27;p<0.001) were proven to have strong statistical significance. Similarly, Hypotheses 2 and 3 were all supported because the four paths from job insecurity and organizational pandemic responses to job stress were all statistically significant. Next, Hypothesis 4 proposed significant links between pandemic fear and coping behaviors, which were supported. In addition, job insecurity merely had a significant effect on emotion-focused coping, while organizational pandemic response merely had a significant effect on problem-focused coping. Therefore, Hypotheses 5 and 6 were partially supported. Further, Hypotheses 7–9 proposed that coping behaviors mediated the relationships between pandemic perception and job stress. The results indicated that only Hypotheses 7a and 9a were supported.

## 5. Discussion

Construction is generally accepted as one of the most stressful industries due to its demanding tasks and high-risk environment [18]. During the COVID-19 pandemic, greatly changed working conditions (e.g., delayed and suspended construction projects, epidemic prevention measures, etc.) may have caused higher job stress. This research investigated the effect of pandemic perception on job stress by considering coping behaviors as the mediators. To the best of our knowledge, this research is one of the few to explore the impact of pandemic perception on job stress among construction workers during the current COVID-19 pandemic. The findings provide insights into how to alleviate pandemic impacts as well as improve the well-being of construction workers.

### 5.1. Main Findings

In line with other related studies, this research found that pandemic perception was significantly related to job stress [65,66,67]. It indicated that construction workers who experienced high levels of fear of COVID-19 and job insecurity were more likely to report an increased level of psychological and physical stress symptoms. Fear of being infected and job loss are the two most common sources of stress experienced among construction workers during the current pandemic. This is not surprising because most construction activities must be performed physically onsite, where construction workers work together at close range, and are at high risk of being exposed to COVID-19 [68]. In addition, delays, suspensions, and cancelations of construction projects due to workforce shortage, disruption in supply chains, and lockdowns have caused rising uncertainty and worries for job prospects among construction workers. According to the conservation of resources theory [32], pandemic fear and job insecurity may consume individual time and energy, which eventually increases their stress symptoms. By contrast, the findings indicated that organizational pandemic responses, as critical job resources during the pandemic, could alleviate construction workers’ job stress.

Furthermore, according to the magnitude of the path coefficients, it was found that compared with job insecurity (β=0.15;p<0.05) and organizational pandemic responses (β=−0.13;p<0.05), pandemic fear had a much stronger effect on psychological stress (β=0.27;p<0.001). This may be explained that pandemic fear can stimulate negative psychological reactions more frequently than other aspects of pandemic perception. The results also indicated that job insecurity had a much stronger effect on physical stress  (β=0.25;p<0.001) than psychological stress (β=0.15;p<0.05). This could be explained by the job preservation mechanism, where individuals tend to work much harder when perceiving a threat of job loss [69].

This research found that different perceptions of the current pandemic could trigger different types of coping behaviors. Specifically, pandemic fear and job insecurity were more likely to increase emotion-focused coping, while organizational pandemic response was more likely to increase problem-focused coping. Construction workers often have limited education levels and are often placed in relatively unfavorable positions in the hierarchical structure of any construction organizations [70]. When encountering pandemic impacts and job insecurity, they tend to adopt emotion-focused coping because they have limited resources and methods to solve problems that cause stress [71]. Nevertheless, the significant relationship between organizational pandemic response and problem-focused coping indicated that a supportive working environment could motivate workers to deal with stressful situations proactively. Furthermore, this research revealed that problem-focused coping could alleviate both psychological and physical stress. This strengthened the general conclusion that problem-focused coping was adaptive and involved efforts to change a problematic situation to reduce perceived stress [17]. However, this research found that emotion-focused coping could not predict psychological or physical stress. This indicates that the prevalent emotion-focused coping among construction workers was not an appropriate coping strategy to address stress during the pandemic.

### 5.2. Theoretical and Practical Implications

The present research has theoretical implications for both pandemic prevention and occupational health research in the construction context. First, previous studies in general industries (e.g., healthcare and hotel) have confirmed that the current COVID-19 pandemic has negative effects on occupational health of the workforce [65,66,67]. However, construction-related literature primarily focused on the economic, operational, and financial impacts of the pandemic at the project or industry level (e.g., project delays and cost overruns). This research was one of the first to explore pandemic impacts on occupational health (represented by job stress) from the perspective of construction workers. Second, although adverse pandemic impacts have been well-recognized in previous COVID-19-related studies, the mediating mechanism is still not clear. This research could extend the previous studies by considering coping behaviors as the mediators between pandemic perception and job stress. This could provide a better understanding of how pandemic perception stimulates both psychological and physical stress during COVID-19.

This research has practical implications for managers to promote health and safety of construction workers during and after the pandemic. First, considering that pandemic fear and job insecurity could undermine construction workers’ well-being, managers should take measures to alleviate these negative outcomes. To reduce fear of COVID-19, managers should disseminate authentic, updated, and reliable pandemic information through various channels, such as regular health trainings, social media, and physical posters [72,73,74]. To decrease workers’ worries about job loss, managers could give a clear commitment to job prospects. In addition, moderately increased salary levels are also recommended for workers during the pandemic to compensate for the losses induced by project suspensions. Second, this research found that the effect of organizational pandemic response took place through problem-focused coping. Therefore, managers should provide necessary resources such as personal protective equipment, health screening, and vaccination, which are expected to motivate workers to proactively deal with perceived threats and thus decrease their stress [17].

## 6. Conclusions

This research proposed and tested a stressor–coping–stress model to understand the effects of pandemic perception on job stress during the current COVID-19 pandemic. Both problem-focused coping and emotion-focused coping were considered as mediators. This was one of the first studies to explore the pandemic impacts on occupational health and safety among construction workers. The results indicated that pandemic perception had significant effects on both physical and psychological stress. Pandemic fear and job insecurity were more related to emotion-focused coping, while organizational pandemic response was more related to problem-focused coping. Problem-focused coping mediated the relationships between pandemic fear and organizational pandemic response and job stress. These findings could provide meaningful insights for managers in promoting health and well-being of construction workers through distinguishing different pandemic impacts on job stress.

Although sufficient findings have been drawn from the analysis, restrictions cannot be ignored. First, caution needs to be taken in making causal inferences regarding the hypothetical relationships, due to the cross-sectional nature of the data. A longitudinal or experimental design is suggested in the future to validate the findings through much stronger causal inferences. Second, we used the shortened scales of coping behaviors because of the overall length of the survey and concerns about inducing mental fatigue or respondent disengagement. The different dimensions of problem-focused coping (e.g., confrontive coping, planful problem solving, positive reappraisal, etc.) and emotion-focused coping (e.g., seeking emotional support, avoidance, etc.) could be explored in future studies [51]. Third, although the sample in this study was representative, the data were merely collected from twenty-one projects in three Chinese provinces. Therefore, a larger sample is needed to test the generality of the results in the current study. Finally, only the totality of construction workers was the object of this research, and construction workers of different incomes, different types, and different demographic characteristics should be investigated further.

## Figures and Tables

**Table 1:** Results of reliability and convergent validity for variables.

Constructs	Items	SFL	CR	AVE	Cronbach’s Alpha
Pandemic fear	PF1	0.65	0.79	0.43	0.78
	PF2	0.70			
	PF3	0.69			
	PF4	0.59			
	PF5	0.63			
Organizational pandemic response	OPR1	0.80	0.90	0.75	0.90
	OPR2	0.94			
	OPR3	0.85			
Job insecurity	JI1	0.81	0.89	0.74	0.89
	JI2	0.92			
	JI3	0.84			
Problem-focused coping	PFC1	0.71	0.83	0.62	0.82
	PFC2	0.89			
	PFC3	0.76			
Emotion-focused coping	EFC1	0.83	0.876	0.68	0.86
	EFC2	0.92			
	EFC3	0.71			
Physical stress	PHS1	0.86	0.92	0.69	0.90
	PHS2	0.84			
	PHS3	0.79			
	PHS4	0.82			
Psychological stress	PSS1	0.88	0.91	0.77	0.91
	PSS2	0.88			
	PSS3	0.88			

Note: Abbreviations: SFL = Standardized factor loading; CR = Composite reliability; AVE = Average variance extracted.

**Table 2:** Results of discriminant validity.

No.	Constructs	1	2	3	4	5	6	7
1.	Pandemic fear	0.65						
2.	Organizational pandemic response	0.15 **	0.87					
3.	Job insecurity	0.53 ***	0.31 ***	0.86				
4.	Problem-focused coping	−0.04 (n.s.)	0.49 ***	0.13 *	0.79			
5.	Emotion-focused coping	0.36 ***	0.09 (n.s.)	0.31 ***	0.07 (n.s.)	0.82		
6.	Physical stress	0.37 ***	−0.12 *	0.32 ***	−0.23 ***	0.18 ***	0.83	
7.	Psychological stress	0.37 ***	−0.13 *	0.26 ***	−0.23 ***	0.22 ***	0.83 ***	0.88

Note: (1) Correlations are below the diagonal, and the figures in bold on the diagonal are the square root of the AVE of associated constructs. (2) *** *p* < 0.001, ** *p* < 0.01, * *p* < 0.05, n.s. *p* > 0.05.

## Data Availability

The data presented in this study are available on request from the corresponding author.
